# Comparison of outcomes in neck pain patients with and without dizziness undergoing chiropractic treatment: a prospective cohort study with 6 month follow-up

**DOI:** 10.1186/2045-709X-21-3

**Published:** 2013-01-07

**Authors:** B Kim Humphreys, Cynthia Peterson

**Affiliations:** 1 University of Zürich and Orthopaedic University Hospital Balgrist, Forchstrasse 340, 8008, Zürich, Switzerland

**Keywords:** Neck pain, Dizziness, Chiropractic, Outcomes

## Abstract

**Background:**

The symptom ‘dizziness’ is common in patients with chronic whiplash related disorders. However, little is known about dizziness in neck pain patients who have not suffered whiplash. Therefore, the purposes of this study are to compare baseline factors and clinical outcomes of neck pain patients with and without dizziness undergoing chiropractic treatment and to compare outcomes based on gender.

**Methods:**

This prospective cohort study compares adult neck pain patients with dizziness (n = 177) to neck pain patients without dizziness (n = 228) who presented for chiropractic treatment, (no chiropractic or manual therapy in the previous 3 months). Patients completed the numerical pain rating scale (NRS) and Bournemouth questionnaire (BQN) at baseline. At 1, 3 and 6 months after start of treatment the NRS and BQN were completed along with the Patient Global Impression of Change (PGIC) scale. Demographic information was also collected. Improvement at each follow-up data collection point was categorized using the PGIC as ‘improved’ or ‘not improved’. Differences between the two groups for NRS and BQN subscale and total scores were calculated using the unpaired Student’s t-test. Gender differences between the patients with dizziness were also calculated using the unpaired t-test.

**Results:**

Females accounted for 75% of patients with dizziness. The majority of patients with and without dizziness reported clinically relevant improvement at 1, 3 and 6 months with 80% of patients with dizziness and 78% of patients without dizziness being improved at 6 months. Patients with dizziness reported significantly higher baseline NRS and BQN scores, but at 6 months there were no significant differences between patients with and without dizziness for any of the outcome measures. Females with dizziness reported higher levels of depression compared to males at 1, 3 and 6 months (p = 0.007, 0.005, 0.022).

**Conclusions:**

Neck pain patients with dizziness reported significantly higher pain and disability scores at baseline compared to patients without dizziness. A high proportion of patients in both groups reported clinically relevant improvement on the PGIC scale. At 6 months after start of chiropractic treatment there were no differences in any outcome measures between the two groups.

## Introduction

The complaint of neck pain is second only to low back pain in terms of common musculoskeletal problems in society today with a lifetime prevalence of 26-71% and a yearly prevalence of 30-50% [1,2]. Most concerning is that many patients, particularly those in the working population or who have suffered whiplash trauma, will become chronic and continue to report pain and disability for greater than 6-months [3-6]. In terms of symptoms, dizziness and unsteadiness are the most frequent complaints following pain for chronic whiplash sufferers with up to 70% of patients reporting these problems [7,8]. Apart from whiplash trauma, little is known about dizziness in the chronic neck pain population and much remains unknown about the etiology of chronic neck pain in general [9].

Gender differences in reporting pain intensity is currently a topic of debate. Recent research suggests that females report more pain because they feel pain more intensely than males over a variety of musculoskeletal complaints [10,11]. Furthermore, LeResche suggests that these differences may not be taken into account by health care providers, leading to less than optimal pain management for females [12]. However gender differences in neck pain patients with or without dizziness have not been described with respect to clinical outcomes over time.

Therefore, the purposes of this study on neck pain patients receiving chiropractic care are twofold: 1. to compare baseline variables and the clinical outcomes of neck pain patients with and without dizziness in terms of clinically relevant ‘improvement’, pain, disability, and psychosocial variables over a 6-month period; 2. to evaluate gender differences for neck pain patients with dizziness in terms of clinically relevant ‘improvement’, pain, disability, and psychosocial variables in a longitudinal study.

## Methods

This is a prospective cohort study with 6 month follow-up. Ethics approval was obtained from the Orthopaedic University hospital Balgrist and Kanton of Zürich, Switzerland ethics committees (EK-19/2009) and written informed consent was obtained from all patients.

### Patients

Consecutive new patients over the age of 18 with neck pain of any duration who had not undergone chiropractic or manual therapy in the prior 3 months were recruited from multiple chiropractic practices in Switzerland. All 280 members of the Swiss Association for Chiropractic were invited to participate in the study and 81 practitioners from both the German and French geographic regions of Switzerland chose to enrol patients. There were no set number of patients required from participating clinicians and all chiropractors were strongly encouraged during meetings and with frequent e-mail reminders to enrol all qualifying patients. Patients with specific abnormalities of the cervical spine that are contraindications to chiropractic manipulative therapy, such as tumours, infections, inflammatory arthropathies, acute fractures, Paget’s disease, cervical spondylotic myelopathy, known unstable congenital anomalies and severe osteoporosis, were excluded. Additionally, patients on anticoagulation therapy were also excluded.

### Demographic and baseline data

Information provided by the treating chiropractor at the initial consultation included: patient age, gender, marital status, whether or not the onset of pain was due to trauma, whether or not the patient smokes, whether or not the patient was currently taking pain medication, duration of current complaint, number of previous episodes, whether or not the patient also complained of dizziness and the patient’s general health status (good, average or poor). This information was completed on a baseline information form. For dizziness, patients were asked to report if they currently experienced ‘dizziness’ which was described as feelings of ‘light-headedness’ or faintness or disorientation or unsteadiness or reduced postural and balance control that was related to their neck pain.

The eleven point numerical rating scale (NRS) for current neck pain ( 0 = no pain, 10 = the worst pain imaginable) and a separate NRS for current arm pain as well as the Bournemouth Questionnaire for neck (BQN) disability, were administered to the patient immediately prior to the first treatment by the office staff of each practice. The BQN is a multidimensional instrument covering 7 domains with each domain evaluated using an 11-point numerical rating scale (0 through 10). The seven domains include: (i) pain; (ii) disability (activities of daily living (ADL)); (iii) disability (social activities); (iv) anxiety; (v) depression; (vi) work, both inside and outside the home, fear avoidance; and (vii) locus of control. Each domain is evaluated independently on an 11 point scale with 0 indicating ‘not at all affected’ and ‘10’ indicating ‘maximally affected’. In addition to each domain score, the total score (maximum 70 points) is also calculated. The BQN has been translated and validated in both German and French with the seven domains as well as the over-all score having been shown to be more sensitive to change in a patient’s condition compared to other similar outcome measures [13,14].

### Outcome measures

#### Primary outcome

Telephone interviews were conducted 1, 3 and 6 months after the first chiropractic treatment to collect the outcome data. The primary outcome of ‘improvement’ for both neck pain and the symptom of ‘dizziness’ was evaluated using the Patient’s Global Impression of Change (PGIC) scale [15] for the neck pain as well as a PGIC scale specifically concerned with dizziness. The PGIC is a 7 point scale ranging from ‘much better’, ‘better’, slightly better’, no change’, slightly worse’, ‘worse,’ and ‘much worse’. Only the responses of ‘much better’ and ‘better’ were considered clinically relevant improvement [16,17].

#### Secondary outcomes

**Figure 1:**
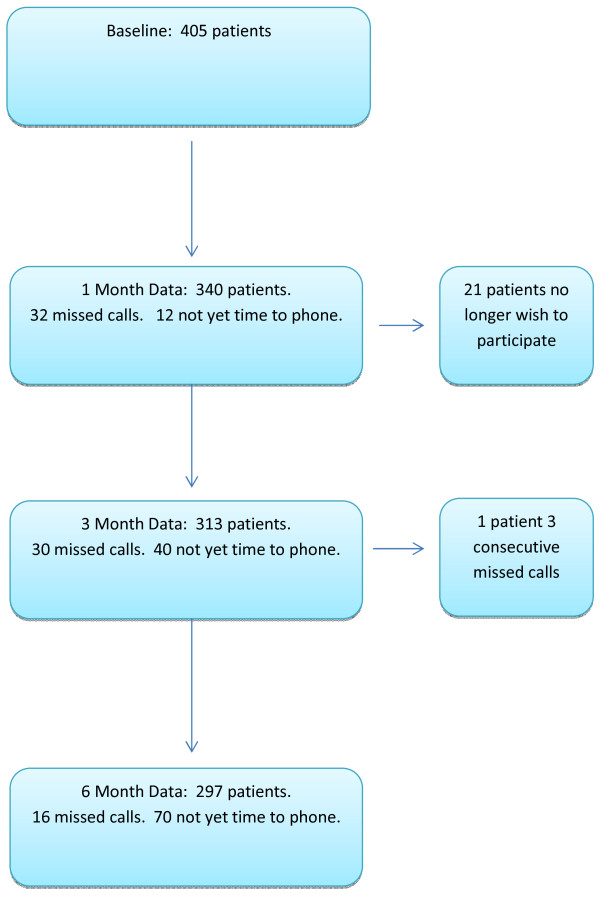
Flow chart showing number of patients with data at the various data collection time points.

Additionally, data from the NRS (neck), NRS (arm), and the BQN were also collected as secondary outcome measures at 1 month, 3 months and 6 months after the start of treatment via telephone interviews (Figure 1). These telephone interviews were conducted by research assistants at the university hospital who were unknown to the patients.

### Statistical analysis

Evaluation of differences between the patients with and without dizziness for the demographic categorical variables was done using the Chi-squared test. Differences between the patient ages and the baseline NRS and BQN subscale and total scores for the two groups were calculated using the unpaired Student’s t-test. The proportion of patients with and without dizziness reporting ‘improvement’ for their neck pain on the PGIC scale was calculated at each data collection time point. For those patients with dizziness, a separate PGIC was used to report ‘improvement’ specifically for their dizziness complaint [15]. The Chi-squared test was used to assess differences in the proportions of patients ‘improved’ for their neck pain between these two groups.

Evaluation of the frequency distributions of the NRS and BQN subscale and total scores was done to determine whether or not they were normally distributed. As they were determined to be quite normally distributed, assessment of differences between the NRS and BQN subscale and total scores as well as the change scores for the two groups at all follow-up time periods was calculated using the unpaired Student’s t-test. The Mann Whitney U test for non-parametric numerical data was used to compare the follow-up mean PGIC scores between the two groups. Within group comparisons between the baseline NRS and BQN scores and outcomes at all time points were done using the paired t-test.

Differences between males and females for categorical baseline variables, including the presence or absence of ‘dizziness’, were assessed using the Chi-squared test. Comparison of the genders for age, baseline NRS (neck), NRS (arm), BQN subscale and BQN total scores were done using the unpaired Student’s t-test. Male and female patients with and without dizziness were compared for the proportion reporting ‘improvement’ on the PGIC scale at 1, 3 and 6 months and the Chi-squared test was used to investigate a gender difference in ‘improvement’. The unpaired t-test was also used to compare the follow-up NRS and BQN scores between the genders. Within gender differences in follow-up NRS and BQN scores compared to baseline scores were assessed using the paired t-test.

## Results

### Demographic information

Four hundred and five neck pain patients with baseline data who had consented to be part of the Chiropractic Outcome Study in Switzerland were included in this study. Eighty-one or 29% of the 280 members of the Association of Swiss Chiropractors recruited patients for this study from the two largest geographic regions of Switzerland (German and French). Of the 405 patients, 177 (44%) reported neck pain and related dizziness while 228 reported that they had neck pain without dizziness. Baseline demographic factors comparing patients with and without dizziness are shown in Table 1. A significant majority of the patients with dizziness were female. In addition to being female (p = 0.001), neck pain patients with dizziness were more likely to be smokers (p = 0.04).

**Table 1:** Comparison of neck pain patients undergoing chiropractic treatment with and without dizziness at baseline

	**Dizziness Present (N = 177)**	**Dizziness Absent (N = 226)**	**P value**
Gender: % Female	75%	58%	**0.001**
Percentage Smokers: Yes	27%	18%	**0.04**
Pain medication: Yes	28%	30%	0.74
Trauma onset: Yes	15%	19%	0.32
Duration of complaint: Acute =< 4 Wks	35%	38.%	0.71
Subacute = 4 – 12 Wks	19.%	19%	
Chronic = > 12 Wks	46%	44%	
General Health:	Good = 54%	Good = 61%	0.35
	Average = 41%	Average = 35%	
	Poor = 5%	Poor = 4%	
Previous Episodes:			
None	43%	45%	0.65
1-3	15%	16%	
4 or More	41%	39%	
	**Mean (+/−SD) (N = 177)**	**Mean (+/−SD) (N = 226)**	
Age	41.3 (14.1)	42.9 (14.1)	0.28
Baseline Neck Pain NRS score (current)	6.3 (2.4)	5.7 (2.3)	**0.011**
Baseline Arm Pain NRS Score	2.9 (3.1)	1.8 (2.2)	**0.0001**
BQN 1 Pain (average past week)	6.0 (2.4)	5.6 (2.3)	0.052
BQN 2 Disability (ADL)	4.9 (3.0)	3.9 (2.7)	**0.001**
BQN 3 Disability (social activities)	4.2 (3.1)	3.2 (3.0)	**0.002**
BQN 4 Anxiety	6.3 (2.5)	5.2 (2.8)	**0.0001**
BQN 5 Depression	4.8 (3.1)	2.9 (3.0)	**0.0001**
BQN 6 Work, Fear Avoidance	5.4 (2.9)	4.2 (3.0)	**0.0001**
BQN 7 Locus of Control	5.4 (2.8)	4.7 (2.9)	**0.03**
BQN Total	36.4 (15.6)	29.9 (14.7)	**0.0001**

NRS = numerical rating scale for pain. ADL = activities of daily living. SD = standard deviation. N = number of patients. WKs = Weeks.

### Baseline pain and disability differences

At baseline neck pain patients with dizziness reported statistically significantly higher levels of neck pain, arm pain, physical and social disability, anxiety, depression, work fear avoidance and less control over their pain condition (locus of control) compared to those without dizziness. As a result, the BQN total scores were significantly different between those with and without dizziness, being significantly higher in neck pain patients with dizziness (p = 0.0001) (Table 1).

### Primary outcome - clinically relevant ‘Improvement’

There was a steady report of increased improvement for both neck pain and dizziness at each of the three follow-up data collection periods with no differences in outcome between patients with and without dizziness (Table 2). Tables 3, 4 and 5 show the actual PGIC mean scores (+/− standard deviations) for the two groups.

**Table 2:** Comparison of neck pain patients with and without dizziness undergoing chiropractic care in terms of clinically relevant ‘improvement’ for neck pain as well as the dizziness in this prospective outcomes study

	**Dizziness Present (N = 177)**	**Dizziness Absent (N = 228)**
**1 Month % ‘Improved’ for neck pain**	72%	73%
**1 Month % ‘Improved’ for Dizziness**	50%	
**3 Months % ‘Improved’ for neck pain**	81%	81%
**3 Months % ‘Improved’ for Dizziness**	81%	
**6 Months % ‘Improved’ for neck pain**	80%	78%
**6 Months % ‘Improved’ for Dizziness**	80%	

**Table 3:** 1 Month mean PGIC, NRS and NRS change scores comparing neck pain patients with and without dizziness who received chiropractic treatment in this prospective outcomes study

	**Dizziness Present (N = 141)**	**Dizziness Absent (N = 199)**	**P value**
	***Mean Change (***Δ***) Score (95% CI)*Mean Scale Score (+/−SD)**	***Mean Change (Δ)Score (95% CI)*Mean Scale Score (+/−SD)**	
1 Month PGIC (neck pain) score	1.9 (1.2)	2.0 (1.2)	0.85
1 Month PGIC (dizziness) score	2.1 (1.3)	2.3 (1.4)	0.39
1 Month Neck Pain NRS Δ score (current)	***3.1 (2.6-3.9)***	***2.8 (2.4-3.2)***	***0.33***
1 Month mean score	3.1 (2.2)	2.9 (2.4)	0.34
1 Month Arm Pain NRS Δ score	***1.4 (0.9-1.9)***	***0.8 (0.5-1.1)***	0.04*
1 Month mean score	1.6 (2.3)	1.0 (1.8)	0.003*
BQN 1 Pain Δ (average past week)	***2.7 (2.8-3.2)***	***2.7 (2.3-3.0)***	***0.96***
BQN 1 mean score	3.3 (2.17)	2.8 (2.2)	0.09
BQN 2 Disability Δ (ADL)	***2.6 (2.1-3.1)***	***2.1 (1.7-2.6)***	***0.21***
BQN 2 mean score	2.1 (2.5)	1.6 (2.4)	0.08
BQN 3 Disability Δ (social activities)	***2.5 (1.9-3.1)***	***2.0 (1.6-2.5)***	***0.20***
BQ 3 mean score	1.6 (2.5)	1.1 (2.3)	0.03*
BQN 4 Anxiety Δ	***3.0 (2.5-3.6)***	***2.7 (2.3-3.2)***	***0.37***
BQN 4 mean score	3.0 (2.9)	2.5 (2.7)	0.06
BQN 5 Depression Δ	***2.8 (2.3-3.4)***	***1.6 (1.2-2.0)***	0.001*
BQN 5 mean score	1.9 (2.7)	1.2 (2.2)	0.02*
BQN 6 Work, Fear Avoidance Δ	***2.4 (1.9-3.0)***	***1.4 (0.7-2.1)***	0.03*
BQN mean score	2.7 (2.8)	2.8 (4.1)	0.94
BQN 7 Locus of Control Δ	***2.0 (1.4-2.6)***	***1.2 (0.7-1.8)***	***0.06***
BQN 7 mean score	3.4 (2.8)	3.4 (3.4)	0.97
BQN Total Δ	***17.6 (14.7-20.6)***	***14.2 (11.8-16.5)***	***0.08***
BQN Total mean score	17.8 (14.5)	15.1 (13.8)	0.09

NRS = numerical rating scale for pain. Δ = Change. ADL = activities of daily living. PGIC = patient’s global impression of change. CI = Confidence Intervals. SD = standard deviation. N = number of patients. * = p < 0.05.

**Table 4:** 3 Months mean PGIC, NRS and NRS change scores comparing neck pain patients with and without dizziness who received chiropractic treatment in this prospective outcomes study

	**Dizziness Present (N = 129)**	**Dizziness Absent (N = 184)**	**P value**
	***Mean Change (***Δ***) Score (95% CI)*Mean Scale Score (+/−SD)**	***Mean Change (***Δ***) Score (95% CI)*Mean Scale Score (+/−SD)**	
3 Months PGIC (neck pain) score	1.8 (1.2)	1.7 (1.1)	0.64
3 Months PGIC (dizziness) score	1.8 (1.3)	2.0 (1.6)	0.51
3 Months Neck Pain NRS Δ score (current)	***3.5 (3.0-4.0)***	***3.5 (3.1-3.9)***	***0.88***
3 Months mean score	2.5 (2.4)	2.3 (2.2)	0.30
3 Months Arm Pain NRS Δ score	***1.7 (1.2-2.3)***	***1.1 (0.8-1.5)***	***0.06***
3 Months mean score	1.0 (1.7)	0.8 (1.8)	0.27
BQN 1 Pain Δ (average past week)	***3.1 (2.6-3.7)***	***3.2 (2.8-3.6)***	***0.75***
BQN1 mean score	2.6 (2.3)	2.4 (2.3)	0.30
BQN 2 Disability Δ (ADL)	***3.4 (2.8-4.0)***	***2.8 (2.3-3-2)***	***0.07***
BQN 2 mean score	1.3 (2.0)	1.1 (1.9)	0.31
BQN 3 Disability Δ (social activities)	***3.1 (2.5-3.7)***	***2.8 (2.4-3-2)***	***0.41***
BQN 3 mean score	0.8 (1.9)	0.4 (1.4)	0.03*
BQN 4 Anxiety Δ	***3.9 (3.3-4.5)***	***3.5 (3.0-4.0)***	***0.31***
BQN 4 mean score	2.3 (2.6)	1.7 (2.4)	0.07
BQN 5 Depression Δ	***3.3 (2.7-3.9)***	***2.2 (1.8-2.6)***	0.003*
BQN 5 mean score	1.4 (2.3)	0.8 (1.7)	0.02*
BQN 6 Work, Fear Avoidance Δ	***3.1 (2.5-3.7)***	***2.2 (1.7-2.6)***	0.03*
BQN 6 mean score	2.0 (2.7)	2.0 (2.7)	0.97
BQN 7 Locus of Control Δ	***2.8 (2.1-3.5)***	***2.1 (1.6-2.7)***	***0.14***
BQN 7 mean score	2.6 (2.9)	2.7 (3.1)	0.69
BQN Total Δ	***22.2 (18.8-25.5)***	***19.3 (16.9-21-6)***	***0.16***
BQN Total mean	12.8 (13.2)	11.0 (11.3)	0.17

NRS = numerical rating scale for pain. Δ = Change. ADL = activities of daily living. PGIC = patient’s global impression of change. CI = Confidence Intervals. SD = standard deviation. N = number of patients. * = p < 0.05.

**Table 5:** 6 Months mean PGIC, NRS and NRS change scores comparing neck pain patients with and without dizziness who received chiropractic treatment in this prospective outcomes study

	**Dizziness Present (N = 121)**	**Dizziness Absent (N = 176)**	**P value**
	***Mean Change (***Δ***) Score (95% CI)*Mean Scale Score (+/−SD)**	***Mean Change (***Δ***) Score (95% CI)*Mean Scale Score (+/−SD)**	
6 Months PGIC (neck pain) score	1.7 (1.1)	1.7 (1.2)	0.93
6 Months PGIC (dizziness) score	1.8 (1.0)	1.9 (1.2)	0.42
6 Months Neck Pain NRS Δ score (current)	***3.6 (3.1-4.2)***	***3.6 (3.2-4.0)***	***0.83***
6 Months mean score	2.5 (2.4)	2.1 (2.2)	0.10
6 Months Arm Pain NRS Δ score	***1.9 (1.3-2.4)***	***1.3 (1.0-1.6)***	***0.07***
6 Months mean score	1.0 (2.1)	0.6 (1.6)	0.07
BQN 1 Pain Δ (average past week)	***3.3 (2.8-3.8)***	***3.3 (2.9-3.7)***	***0.90***
BQN 1 mean score	2.5 (2.4)	2.2 (2.2)	0.30
BQN 2 Disability Δ (ADL)	***3.6 (3.0-4.1)***	***2.7 (2.3-3.2)***	0.02*
BQN 2 mean score	1.2 (1.8)	1.0 (2.0)	0.56
BQN 3 Disability Δ (social activities)	***3.2 (2.7-3.8)***	***2.5 (2.0-3.0)***	***0.05***
BQN 3 mean score	0.8 (1.7)	0.6 (1.8)	0.40
BQN 4 Anxiety Δ	***4.2 (3.6-4.8)***	***3.3 (2.8-3.8)***	0.04*
BQN 4 mean score	2.0 (2.5)	1.9 (2.4)	0.68
BQN 5 Depression Δ	***3.5 (2.8-4.1)***	***2.0 (1.6-2.5)***	0.0001*
BQN 5 mean score	1.1 (2.1)	0.9 (2.5)	0.41
BQN 6 Work, Fear Avoidance Δ	***3.0 (2.4-3.7)***	***2.3 (1.8-2.8)***	***0.09***
BQN 6 mean score	2.1 (2.8)	1.9 (2.5)	0.58
BQN 7 Locus of Control Δ	***3.2 (2.5-3.9)***	***2.2 (1.7-2.7)***	0.02*
BQN 7 mean score	2.3 (2.7)	2.6 (3.0)	0.40
BQN Total Δ	***23.6 (20.4-26.9)***	***18.7 (16.3-21.1)***	0.02*
BQN Total mean score	11.9 (12.7)	11.1 (12.1)	0.59

NRS = numerical rating scale for pain. Δ = Change. ADL = activities of daily living. PGIC = patient’s global impression of change. CI = Confidence Intervals. SD = standard deviation. N = number of patients. * = p < 0.05.

### Secondary outcome differences

By 1 month after the start of treatment, the only areas where the neck pain with dizziness patients reported significantly higher scores were arm pain, social disability and depression (Table 3) but there was no significant difference in the BQN total score between the two groups. Three months after the start of treatment only social disability and depression were scored significantly higher in patients with dizziness (Table 4) and by 6 months there were no significant differences between neck pain patients with and without dizziness for any of the outcome measures due to the significantly higher baseline to 6 month change scores in the BQN subscales ‘physical disability’, ‘anxiety’, ‘depression’ and ‘locus of control’ for patients with dizziness (Table 5).

### Gender differences

There was a significant association between being female and reporting ‘dizziness’ (p = 0.001) with 133 of the 177 patients stating that they had neck pain and dizziness being female (75%). Comparing male and female patients with and without dizziness, there were no significant gender differences for age, duration of complaint, presence of radiculopathy, trauma onset, smoking, general health or baseline report of neck pain or arm pain. Females with and without dizziness reported significantly higher baseline scores for ‘depression’ compared to males with and without dizziness, with the scores being higher in the patients with dizziness compared to those without.

There was no significant difference in the proportion of males and females with and without dizziness who improved at any time point and both genders with and without dizziness reported significantly improved NRS and BQN scores compared to baseline at all follow-up time points. Table 6 shows the percentage of males and females with dizziness who ‘improved’ specifically for their dizziness complaint at each time point.

**Table 6:** Comparison of the proportion of male and female neck pain patients with dizziness who received chiropractic treatment in this prospective outcomes study reporting ‘improvement’ for their ‘dizziness’ at each time point and the specific BQN questions that were significantly different at all data collection time periods

	**Females (N = 133)**	**Males (N = 44)**	**P value**
**1 Month % ‘Improved’ for Dizziness**	69%	61%	0.36
**3 Months % ‘Improved’ for Dizziness**	77%	82%	0.77
**6 Months % ‘Improved’ for Dizziness**	81%	70%	0.34
	**Mean (+/−SD)**	**Mean (+/− SD)**	
**Baseline** BQN5 Depression	4.0 (3.3)	3.3 (3.0)	0.03
**1 Month** BQN 5 Depression	1.7 (2.6)	1.0 (1.9)	0.007
**3 Month** BQN 5 Depression	1.3 (2.2)	0.6 (1.4)	0.005
**6 Month** BQN 5 Depression	1.2 (2.3)	0.6 (1.5)	0.022

SD = standard deviation. N = number of patients. BQN = Bournemouth questionnaire for neck pain and disability.

For those patients *without* dizziness, females reported higher levels of depression only at 3 months compared to males. For patients *with* dizziness however, depression levels continued to be rated significantly differently between the genders at all follow-up time points, with higher scores in female patients (Table 6).

## Discussion

The findings from this study are very encouraging for neck pain patients undergoing chiropractic treatment who also suffer from dizziness. A high proportion of neck pain patients with and without dizziness reported clinically relevant improvement at 1 month, 3 months and 6 months, with 80% of patients with dizziness reporting that they were significantly ‘improved’ specifically relating to their dizziness symptoms at 6 months. Only the scores of ‘much better’ or ‘better’ (1 or 2) were counted as clinically relevant improvement. ‘Slightly better’ was not considered to be improved in order to error on the side of caution [16,17]. Statistically significant decreases in all secondary outcome measures at every data collection time point were also found for both groups, although arm pain was somewhat slower to respond in females. However, the low mean baseline NRS scores both for the patients with and without dizziness shows that compared to other pain, disability and functional measures, arm pain was the least problematic.

It is important to point out that at baseline neck pain patients with dizziness reported significantly higher scores for severity of neck pain, arm pain, all subscales on the BQN as well as the BQN total score compared to the neck pain patients without dizziness. However, over time fewer differences between these two groups were found with no significant differences between the two cohorts at the 6 month data collection time point. Depression and social disability were the two categories that remained significantly different at one and 3 months. However, although significant, the mean scores at 3 months of 1.35 and 0.81 for depression and 0.83 and 0.43 for social disability are very low on the 11 point BQN subscales so it can be suggested that these differences are clinically unimportant. The BQN subscale ‘depression’ stood out as the most dramatic difference between patients with and without dizziness as well as between males and females with and without dizziness. It was nearly 2 points higher in the patients with dizziness at baseline but also demonstrated the most dramatic change score at 6 months of nearly 3.5 points. At that time point the mean score was no longer significantly different compared to patients without dizziness.

It was somewhat surprising to find that nearly 44% of neck pain patients presenting to Swiss chiropractors stated that they had associated dizziness. However, the fact that 75% of neck pain patients with dizziness in this study were female is not surprising. It is well documented that females are more likely to suffer from neck pain in general [10-12] and that a large proportion of chronic whiplash sufferers report symptoms of dizziness and unsteadiness [7,8]. However, what is unusual in this study is that there was no difference between neck pain patients with and without dizziness in terms of a trauma onset.

Cervicogenic dizziness or dizziness of suspected cervical origin with or without unsteadiness can arise from mechanical, degenerative, inflammatory or traumatic problems affecting various structures of the neck [18]. In particular, altered afferent information from dysfunctional mechanoreceptors in the cervical facet joints and deep cervical tissues and neck muscles, especially in whiplash patients, may lead to cervicogenic dizziness [6-8,18,19]. The dizziness and unsteadiness is thought to arise from dysfunction of the cervical somatosensory system [7,8,20]. In particular there is a mismatch of sensory information from the dysfunctional deep cervical tissues and proprioceptors compared to the vestibular and oculomotor afferent impulses [19,20].

Therefore it is hypothesized that manual therapy such as spinal manipulation may be effective in treating cervicogenic dizziness by restoring normal movement of the zygoapophyseal joints, reducing pain and muscle hypertonicity and thereby restoring normal proprioceptive and biomechanical functioning of the cervical spine [18,21]. Indeed, current evidence, although limited, supports a neuroanatomical and neurophysiological basis for cervicogenic dizziness and that manual therapy particularly in the upper cervical spine may be helpful in reducing cervicogenic dizziness [18].

Limitations to this study must start by stating that because this was not a randomized clinical trial the favourable results reported here cannot be attributed to the chiropractic treatment. There was also no attempt to compare outcomes based on the specific treatments applied or the frequency of treatment. Additionally, acute vs. chronic patients were not evaluated separately because no difference in duration of complaint was found between those with and without dizziness. It is well known that most acute neck pain patients improve due to natural history. Neck pain is most likely recurrent however, and as such, the improvement noted by these patients may very well be noteworthy. Another limitation to this study may be that there were fewer patients with 6 month data compared to baseline data. This was primarily due to the fact that this is an ongoing study and the time point had not yet been reached for the 6 month telephone call. However, with 121 patients with baseline dizziness and 176 patients without dizziness at baseline for the 6 month data collection time point, additional patients would be unlikely to alter the results [16].

The fact that only 29% of practicing chiropractors contributed patients to this study may also be a limitation as it is unknown whether or not this sample is representative of the greater chiropractic population. Additionally, some chiropractors contributed several patients and others only a few. It is known however, that chiropractors from the two largest geographic regions of Switzerland submitted patients and that those participating had a wide range of practice experience. Additionally, all Swiss chiropractors must complete a two year full time post-graduate residency programme with a fairly standardized curriculum and pass a rigorous post-graduate examination in order to practice as independent chiropractors in this country. It is known from the Swiss job analysis study published in 2010 that the ‘diversified’ manipulative technique is applied to the majority of patients by the vast majority of chiropractors. Additional commonly applied therapies include trigger point therapy, advice on activities of daily living, therapeutic exercises and mobilization techniques [22]. Thus differences in practices here may be less dramatic than in other countries.

Finally, the use of multiple, uncorrected statistical tests may be another limitation to this study. In particular the large number of statistical tests used in this study may have resulted in a chance-statistically significant finding (one significant finding per 20 tests if p < 0.05). Further exploration of predictors of improvement for neck pain patients with dizziness should use multiple regression analysis.

## Conclusions

A high proportion of patients with and without dizziness reported clinically relevant improvement at 1, 3 and 6 months. Although neck pain patients with dizziness undergoing chiropractic treatment reported significantly higher pain and disability scores at baseline compared to neck pain patients without dizziness, there were no significant differences in any outcome measures between the two groups at 6 months after start of treatment. Neck pain patients with dizziness were much more likely to be female and females with dizziness report higher levels of depression compared to males with dizziness at all data collection time points.

## Competing interests

The authors declare that they have no competing interests.

## Authors’ contributions

BKH: Concept and design of the study, analysis and interpretation of data, drafting and revising the manuscript, final approval of the manuscript. CKP: Collection and entry of data, analysis and interpretation of data, drafting and revising the manuscript, final approval of the manuscript.
